# Role of Dietary Carotenoids in Frailty Syndrome: A Systematic Review

**DOI:** 10.3390/biomedicines10030632

**Published:** 2022-03-09

**Authors:** Roberta Zupo, Fabio Castellana, Sara De Nucci, Annamaria Sila, Simona Aresta, Carola Buscemi, Cristiana Randazzo, Silvio Buscemi, Vincenzo Triggiani, Giovanni De Pergola, Claudia Cava, Madia Lozupone, Francesco Panza, Rodolfo Sardone

**Affiliations:** 1 Unit of Data Sciences and Technology Innovation for Population Health, National Institute of Gastroenterology IRCCS “Saverio de Bellis”, Research Hospital, Castellana Grotte, 70013 Bari, Italy; fabio.castellana@irccsdebellis.it (F.C.); sara.denucci@irccsdebellis.it (S.D.N.); annamaria.sila@irccsdebellis.it (A.S.); arestasimo@gmail.com (S.A.); rodolfo.sardone@irccsdebellis.it (R.S.); 2 Department of Health Promotion, Maternal and Child Health, Internal and Specialty Medicine of Excellence (PROMISE), University of Palermo, 90127 Palermo, Italy; carola.buscemi@gmail.com (C.B.); randazzocristiana@yahoo.it (C.R.); silvio.buscemi@unipa.it (S.B.); 3 Unit of Gastroenterology, Section of Obesity, Metabolic Diseases and Clinical Nutrition, AOU Policlinico “P. Giaccone”, 90127 Palermo, Italy; 4 Section of Internal Medicine, Geriatrics, Endocrinology, and Rare Disease, Interdisciplinary Department of Medicine, School of Medicine, University of Bari, 70124 Bari, Italy; vincenzo.triggiani@uniba.it; 5 Unit of Geriatrics and Internal Medicine, National Institute of Gastroenterology IRCCS “Saverio de Bellis”, Research Hospital, Castellana Grotte, 70013 Bari, Italy; gdepergola@libero.it; 6 Institute of Molecular Bioimaging and Physiology, National Research Council (IBFM-CNR), Via F. Cervi 93, 20131 Milan, Italy; claudia.cava@ibfm.cnr.it; 7 Department of Basic Medicine, Neuroscience, and Sense Organs, University of Bari Aldo Moro, 70124 Bari, Italy; madia.lozupone@gmail.com (M.L.); f_panza@hotmail.com (F.P.)

**Keywords:** carotenoids, physical frailty, aging, adult population, systematic review

## Abstract

Unbalanced diets and altered micronutrient intake are prevalent in the aging adult population. We conducted a systematic review to appraise the evidence regarding the association between single (α-carotene, β-carotene, lutein, lycopene, β-cryptoxanthin) or total carotenoids and frailty syndrome in the adult population. The literature was screened from study inception to December 2021, using six different electronic databases. After establishing inclusion criteria, two independent researchers assessed the eligibility of 180 retrieved articles. Only 11 fit the eligibility requirements, reporting five carotenoid entries. No exclusion criteria were applied to outcomes, assessment tools, i.e., frailty constructs or surrogates, recruitment setting, general health status, country, and study type (cohort or cross-sectional). Carotenoid exposure was taken as either dietary intake or serum concentrations. Cross-sectional design was more common than longitudinal design (*n* = 8). Higher dietary and plasma levels of carotenoids, taken individually or cumulatively, were found to reduce the odds of physical frailty markedly, and the evidence showed consistency in the direction of association across all selected studies. Overall, the methodological quality was rated from moderate (27%) to high (73%). Prevention of micronutrient deficiencies has some potential to counteract physical decline. Considering carotenoids as biological markers, when monitoring micronutrient status, stressing increased fruit and vegetable intake may be part of potential multilevel interventions to prevent or better manage disability.

## 1. Introduction

Preventing geriatric syndromes is critical to enhancing the number of healthy life years in older people as the global population ages. Falls, disability, morbidity, institutionalization, and mortality are all related to physical frailty, a reversible geriatric syndrome affecting 10–15% of community-living older adults [1]. Consistently acknowledged as a markedly reduced physiological reserve of adaptive capacity to cope with stresses [2], frailty affects multiple domains of human functioning. Its multidimensional nature requires a challenging multidisciplinary approach [3,4] across the spectrum of sensory, physical, social, cognitive, oral, psychological/depressive, and nutritional phenotypes [5,6,7,8].

In a multilevel management setting, nutrition has proven to be a viable approach among preventive measures that rely on modifiable factors [9]. Although previous research has focused primarily on the role of weight loss and muscle wasting [10,11,12], the latest findings point to nutritional imbalance as instrumental in shaping frailty risk trajectories, indicating that malnourished, frail older individuals are more likely to become ill and develop multimorbidity, disability, and reduced survival [13]. While poor nutrition, especially a micronutrients imbalance, is common among older individuals [14], the role that micronutrient deficiencies may play in frailty syndrome has not yet been well-characterized. Our previous findings in a Southern Italian older population suggested that combining physical frailty and nutritional imbalance (defined as a high dietary intake of sodium versus a low dietary intake of iron and potassium) doubles the risk of death in such individuals compared to those with either frailty or nutritional imbalance alone [15]. Micronutrient deficiencies have also been associated with many illnesses and factors associated with frailty syndrome, including increased risks of chronic disease [16], impaired immune function [17], reduced antioxidant activity [18], osteoporosis [19], as well as peripheral vascular disease and atherosclerosis [20], and also a more rapid aging process [21].

Extensive research indicates that older adults who follow a Mediterranean-style diet (i.e., a high intake of vegetables, fruits, legumes as the primary source of protein, olive oil as the primary source of lipids, fish as the primary source of animal protein, and grains, nuts, and seeds as the primary source of a variety of nutrients such as vitamin B, omega-3 fatty acids, and antioxidants) have a lower risk of becoming frail within three years [22]. In addition, there is evidence that avoiding a high-fat, low-fiber diet may help prevent or delay frailty in later life.

Among the micronutrients and bioactive compounds introduced in the diet, carotenoids are a group of phytochemicals found abundantly in deep green, yellow, orange, or red fruits and vegetables [23]. Among more than seven hundred structurally defined carotenoids identified in nature, five major elements have been found in human serum, namely α-carotene, β-carotene, lutein, lycopene, and β-cryptoxanthin, that are deemed essential in human nutrition [24].

The potential interaction with physical frailty outcomes [25] opens an explorative window on the role of carotenoids in frailty settings, and conceptual synthesis of the various reports may be useful for risk management purposes. Therefore, the present study systematically evaluates the existing literature on the association between carotenoids and physical frailty in adults. To this end, the following tasks were undertaken: (1) pool all original studies evaluating the impacts of carotenoid (α-carotene, β-carotene, lutein, lycopene, β-cryptoxanthin, and total carotenoids) exposure and physical frailty outcomes in the adult population, (2) evaluate the strength and direction of the association found to finalize our research goal, (3) evaluate the methodological quality and study design, consistency, directionality, precision, size, and (where possible) dose–response gradient of effect estimates in the evidence base according to the Grading of Recommendations, Assessment, Development and Evaluations (GRADE) rating system.

## 2. Methods

### 2.1. Search Strategy and Data Extraction

The present systematic review followed the Preferred Reporting Items for Systematic reviews and Meta-Analyses (PRISMA) guidelines, adhering to the PRISMA 27-item checklist [26]. An a priori protocol for the search strategy and inclusion criteria was established and registered, without particular amendments to the information provided at registration, on PROSPERO, a prospective international register of systematic reviews (CRD42022299910). We looked for original studies in the US National Library of Medicine (PubMed), the Medical Literature Analysis and Retrieval System Online (MEDLINE), EMBASE, Scopus, Ovid, and Google Scholar databases to see if there was a link between carotenoids exposure and physical frailty. Thus, the main objective was to evaluate the association between exposure to different levels of carotenoids, as assessed by dietary intake or plasma concentrations, and physical frailty in the adult population. We also considered the gray literature using the largest archive of preprints https://arxiv.org/ (accessed on 10 January 2022) in the study selection phase and http://www.opengrey.eu/ (accessed on 10 January 2022) database to access conference remarkable conference abstract and other not peer-reviewed material. Since we chose to include only observational studies, the search strategy followed PECO (Populations, Exposure, Comparator, and Outcomes) concepts [27], including populations (adults, at least 30 years of age), exposure (total and individual carotenoids such as α-carotene, β-carotene, lutein, lycopene, β-cryptoxanthin), comparators (exposure levels), and outcomes (physical frailty outcomes). Exposure factors were selected to include five major carotenoids, i.e., α-carotene, β-carotene, lutein, lycopene, and β-cryptoxanthin, regardless of the assessment tool used, hematochemical assay, or dietary intake. Outcomes included physical frailty, regardless of the assessment tool applied, either operationalized (e.g., Cardiovascular Health Study (CHS) phenotype, Frailty Index (FI), and others) or surrogate (e.g., grip/hip/knee strength or gait speed).

The search strategy used in PubMed and MEDLINE and adapted to the other four electronic sources is detailed in Table 1. In the literature search, no time limit was set, and articles were retrieved until 21 December 2021. No language limitation was introduced. Two researchers (R.Z., F.C.) searched the papers, reviewed titles and abstracts of articles retrieved separately and in duplicate, checked full texts, and selected the articles for inclusion in the study. Technical reports, letters to the editor, and systematic and narrative review publications were all omitted. The statistic was used to determine inter-coder agreement, whereas inter-rater reliability (IRR) was used to estimate accuracy and precision. All data extraction stages, both according to PRISMA ideas and coupled with the quality evaluation procedures, yielded a coefficient k of at least 0.9 [28].

### 2.2. Inclusion Criteria, Data Extraction, and Registration

Exposure and outcome had to be referred to an adult population (at least 30 years of age). No criterion was applied to the recruitment settings (hospital, community, or home care) or health status of the study population (general population or groups with specific characteristics). Potentially eligible articles were identified by reading the abstract and, if eligible, then the full-text version of the articles. For each article selected, the best statistical approach with respect to confounding, as applied in evaluating the magnitude of the effect size for associations, was considered. Data were cross-checked, any discrepancies discussed, and disagreements were resolved by a third investigator (R.S.).

The following information was extracted by two investigators (R.Z., F.C.) separately and in duplicate in a piloted form: (1) general information about single studies (author, year of publication, country, settings, design, sample size, age); (2) type of carotenoid (α-carotene, β-carotene, lutein, lycopene, β-cryptoxanthin, and total carotenoids) exposure; (3) outcome(s) regarding physical frailty, including frailty constructs or surrogate measures; (4) main finding(s); (5) effect size of the association between exposure and outcome.

All references selected for retrieval from the databases were managed with the MS Excel software platform for data collection by a biostatistician (FC). Lastly, data extracted from selected studies and stored in the database were structured as tables of evidence.

### 2.3. Quality Assessment within and across Studies and Overall Quality Assessment

The methodological quality of the included studies was independently appraised by paired investigators (R.Z., F.C.), using the National Institutes of Health Quality Assessment Toolkits for Observational Cohort and Cross-Sectional Studies [29,30]. According to the basic toolkit criteria, studies were given high (excellent), fair (moderate), or bad ratings. This tool includes 14 questions that measure bias risk, type I and type II errors, transparency, and confounding variables, i.e., study question, population, participation rate, inclusion criteria, sample size justification, time of measurement of exposure/outcomes, time frame, levels of the exposure, defined exposure, blinded assessors, repeated exposure, defined outcomes, loss to follow-up, and confounding factors. Items 6, 7, and 13 do not refer to cross-sectional studies, and the maximum possible scores for cross-sectional and prospective studies were 8 and 14, respectively. Disagreements between the two investigators on the methodological quality of the studies included in the review were discussed until a consensus was reached with the support of a third investigator (RS). A modified version of the GRADE grading system was used to assess the overall quality of evidence in the research included in this systematic review [31,32]. The following factors were considered: the strength of association for carotenoid(s) exposure and related physical frailty outcomes, methodological quality/design of the studies, consistency, directedness, precision, size, and (where possible) dose–response gradient of the estimates of effects across the evidence base. According to the GRADE rating system, evidence was assessed as extremely low, low, moderate, and high.

## 3. Results

**Figure 1:**
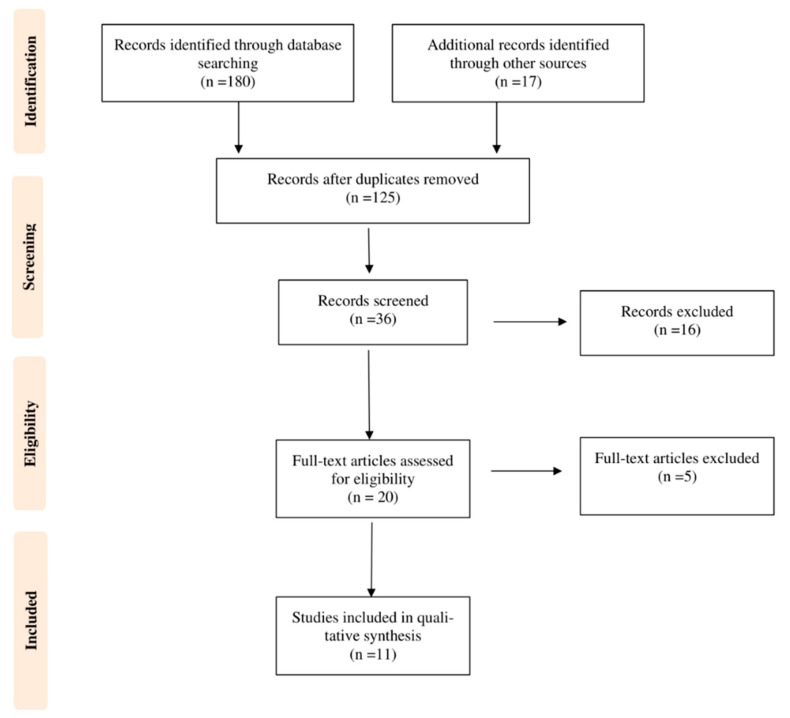
Preferred Reporting Items for Systematic Reviews and Meta-analyses (PRISMA) flow chart illustrating the number of studies at each stage of the review.

The first systematic search of the literature yielded 180 entries. After excluding duplicates, 125 were classified as potentially relevant and selected for title and abstract analysis. Then, 16 were excluded due to not meeting the characteristics of the approach or the review goal. After reviewing the full text of the remaining 36 records, only 11 met the inclusion criterion of age and were included in the final qualitative analysis [33,34,35,36,37,38,39,40,41,42,43]. Figure 1 illustrates the number of studies at each level of the review using the Preferred Reporting Items for Systematic Reviews and Meta-analyses (PRISMA) flow chart. The final study base included 11 articles reporting on five different types of carotenoids. 

Going back to the methodological steps mentioned in the introduction section, we will discuss the results of the studies selected from the existing literature, the strength and direction of the association found, and the methodological quality, in that order.

Details of the design (cohort or cross-sectional), sample size (*n*) and gender ratio (%), minimum age or age range, study population, and country of each study, are provided in Table 2. A descriptive summary reporting differences in plasma carotenoid values by frailty status (presence/absence) across selected studies is provided in Table 3. Cross-sectional (73%, *n* = 8) design was more common than longitudinal design (27%, *n* = 3). Recruitment settings were all community-based, and the geographic distribution of studies was equally distributed between Europe (46%, *n* = 5) and America (54%, *n* = 6). Following the inclusion criteria, subjects were aged over 30, predominantly 65+ years. Among all subjects, gender was skewed toward females, as 3 of the 11 selected studies were entirely female-based. As regards the assessment tool used to evaluate physical frailty, the majority of studies used the CHS phenotype by Fried (*n* = 9), while one study applied two other additional constructs, i.e., the Frailty Index (FI) and the FRAIL Scale, and a minority (*n* = 2) used surrogate measures such as grip/hip/knee strength or gait speed.

**Figure 2:**
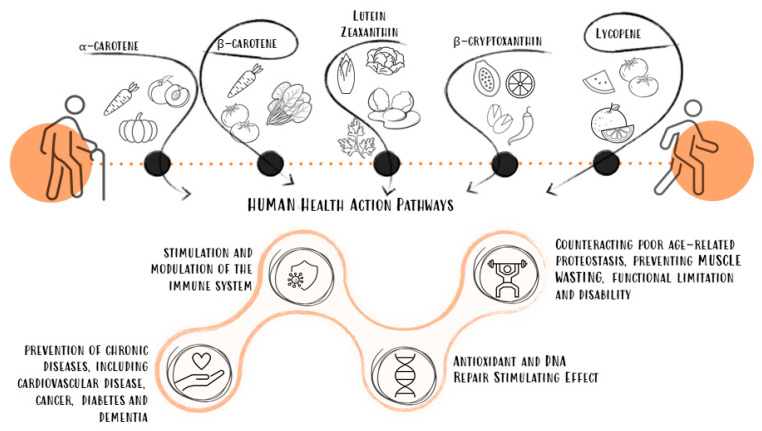
Graphic overview of the findings.

Among the investigated carotenoids, lutein/zeaxanthin (seven studies [34,37,39,40,41,42,43]), α-carotene (seven studies [33,34,37,38,39,41,43]), and β-carotene (seven studies [33,34,37,38,39,41,43]) presented a higher burden of evidence, followed by total carotenoids (five studies [33,34,35,36,43]), β-cryptoxanthin (five studies [33,37,38,39,41]), and lycopene (four studies [37,39,41,43]). Studies investigating plasma concentrations of lutein/zeaxanthin against the presence of physical frailty, in some instances assessing different levels of exposure (tertiles [39] or quartiles [34]), found an inverse relationship; only one study evaluated dietary intake as exposure [43] and found the same direction of the association. The data on α- and β-carotene were very consistent, showing a protective role of high plasma concentrations of these carotenoids on frailty risk, whether assessed by operationalized or surrogate constructs [33,34,37,38,39,41,43]. The four reports on total plasma carotenoids and the one investigating dietary intake followed the same lines, finding a reduced risk of frailty for higher plasma concentrations [33,34,35,36,43] or dietary intake [43]. Then, high plasma levels of β-cryptoxanthin were also protective on the risk of physical frailty in four studies [37,38,39,41], as was lycopene [37,39,41]. Dietary lycopene intake was investigated in a single study and equally described as potentially protective [43]. Lastly, looking at surrogates of physical frailty, high plasma concentrations of lutein/zeaxanthin were protective against progressive loss of grip, hip, and knee strength [42,43]; on the same lines, these performance measures benefited from elevated plasma concentrations of β-cryptoxanthin and total carotenoids [33,34,37,38,39,41,43]. Figure 2 depicts a graphic representation of the findings.

**Figure 3:**
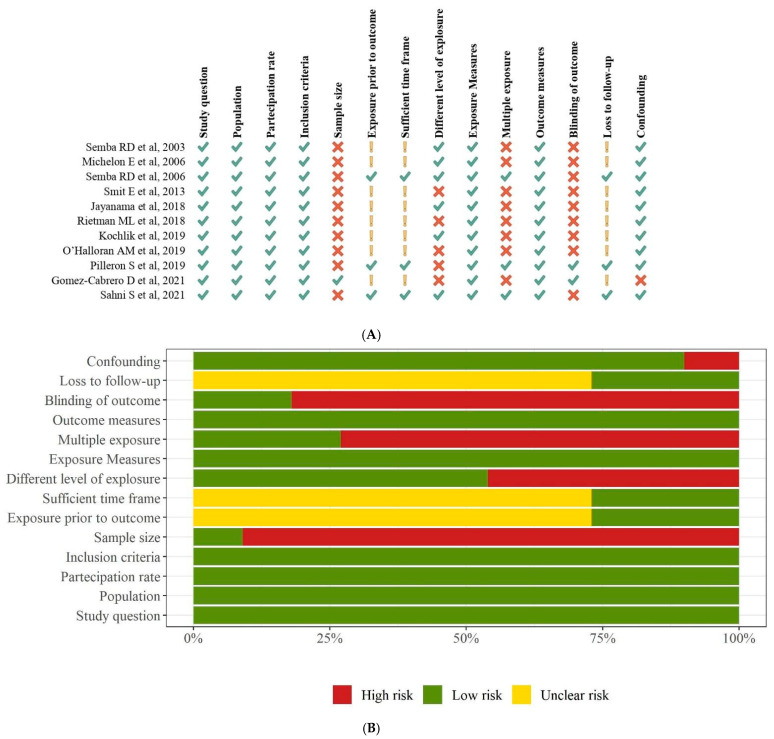
Quality assessment findings using the National Institutes of Health Quality Assessment Toolkits for Observational Cohort and Cross-Sectional Studies. Panel (**A**). Summary of risk of bias. Panel (**B**). Risk of bias graph.

We found moderate (*n* = 3) [36,38,40] to high (*n* = 8) methodological quality among the studies (Table 4). An overview of quality ratings within and across studies is provided in Figure 3 (panel A and B), highlighting areas with higher or lower ratings. Bias was seen primarily in the domains of sample size justification (selection bias) and blinded assessors (detection bias) (91% and 82% of studies, respectively), and to a lesser extent in the domains of the different levels of exposure (46% of studies) and multiple exposure assessments over time (73% of studies). As 73% of the studies had a cross-sectional design, the same percentage reflected an unclear risk for the following qualitative assessment items: exposure prior to the outcome, sufficient time frame, and loss to follow-up.

## 4. Discussion

The present systematic review addressed the conceptual hypothesis of a link between carotenoid deficiency and physical decline in the adult population. To this end, the body of evidence on five major dietary carotenoids (α-carotene, β-carotene, lutein, lycopene, β-cryptoxanthin), as assessed by dietary intake or plasma concentration, was examined against physical frailty outcomes, as evaluated by operationalized constructs or surrogate instruments. The most important finding was the consistency of the direction of association across all studies selected to fill the knowledge gap for the research question. Although most reports had a cross-sectional design, thus leaving little room for causal inference, we found that higher dietary and plasma levels of carotenoids, taken individually or cumulatively, reduced the likelihood of frailty.

As amply acknowledged, physical frailty results from a combination of intrinsic factors, involving changes in molecular and cellular energy levels and extrinsic environmental factors, such as lifestyle [44,45]. Nutritional deficiencies are often overlooked in the clinical setting, although they are shared features among the adult population and gradually worsen in later life. In this context, micronutrients merit special attention, especially in light of accumulated evidence on their human health benefits. Carotenoids are reported to have versatile biological and therapeutic roles [46], including anticancer, immunomodulatory, anti-inflammatory, antibacterial, antidiabetic, and neuroprotective actions [47]. Their ability to counteract oxidative stress caused by the accumulation of reactive oxygen species (ROS) is a common point in many lines of research focused on the preventive management of the aging process and physical decline. Indeed, especially in later life, the body may benefit from antioxidant compounds when antioxidant cellular defense mechanisms are insufficient, and oxidative stress may damage macromolecules such as DNA, proteins, and lipids, causing significant damage to cells and tissues. Along these lines, if the narrow physiological balance between oxidative and antioxidant reactions succumbs to a chronically low intake of dietary antioxidants, such as carotenoids, there will likely be a detrimental effect of ROS on muscle tissue. On this point, the scientific community is confident that a higher intake of antioxidant sources such as carotenoids correlates with improved muscle strength, physical performance, functional limitation, and disability [33,48,49]. Thus, the consumption of fruits and vegetables as primary sources of dietary carotenoids [50], including α-carotene, β-carotene, lutein, lycopene, and β-cryptoxanthin, should be strongly recommended in the adult population. They are especially abundant in yellow-orange fruits (carrots, tomatoes, squash, peppers, among others) and dark green leafy vegetables [51], although masked by chlorophylls. In particular, yellow-orange fruits and vegetables are rich in β-carotene and α-carotene, whereas β-cryptoxanthin, lycopene, and lutein are found in citrus fruits, tomatoes and tomato products, and dark green vegetables, respectively. Egg yolk is a highly bioavailable source of zeaxanthin and lutein.

Although implicated in multiple mechanisms, the primary benefit of carotenoids lies in their antioxidant capacity, as they are very efficient and powerful scavengers of ROS that contribute to oxidative stress. In this respect, high oxidative stress oxidative stress and subsequent inflammation associate with prevalent frailty [48,49], thus it could be assumed that antioxidant properties of diet (such as fruits and vegetables, olive oil, wine, vitamins, and carotenoids) may be involved in protective mechanisms against age-related physical decline. It should be kept in mind that skeletal muscle during everyday aerobic activity sharply increases oxygen uptake, and this inevitably results in increased ROS production. Usually, free radicals produced by mitochondria in working muscle are scavenged by endogenous antioxidants [52]. However, if the buildup of free radicals exceeds antioxidant capacity, a common occurrence in advancing age, the radicals may escape from the mitochondria and oxidize lipids, proteins, sugars, and other cellular components [53]. Based on this assumption, it is likely that increased exposure to, or supplementation of antioxidants such as carotenoids, could improve physical performance while acting to prevent physical frailty.

One further path rests on the evidence that the redox changes toward a high oxidative status in aging, affecting proteostasis [54], that is, protein homeostasis, and skeletal muscle function. As a result, it is likely that proteostasis gives way to the harmful accumulation of protein aggregates and damaged organoids such as mitochondria, among others, leading to loss of skeletal muscle function and mass, and hence a physical decline. In this sense, the oxidation reducing power of carotenoids may counteract this detrimental mechanism. However, a further possible explanation is that an increased consumption of carotenoids may guarantee a better overall diet, more abundant in plant-based foods carrying beneficial phytochemicals, and a better lifestyle [55]. Therefore, the protective effect of carotenoids may be indirect, warranting the conduction of intervention trials to corroborate the association.

Some limitations in the present study should be considered. The limited data and heterogeneity of physical frailty assessment tools lower the quantitative and qualitative reliability. Moreover, designs differed among the selected studies; the cross-sectional design was more common, thus leaving little room for discussion of a causal inference. The statistical method to elucidate the effects (association) of carotenoid exposure on frailty status and the assessment tools used to estimate carotenoid exposure (dietary intake or plasma concentrations) differed across studies. Additionally, the selected studies differed in terms of sample size, and female gender was found to be prevalent overall. Other than this, the novelty of the theme, so far lacking a qualitative synthesis, and the aspect of adjustment for confounding variables taken into account in the vast majority of the selected studies should be considered to further strengthen the resulting associations.

## 5. Conclusions

Preventing micronutrient deficiencies may potentially reduce the risk of disability, although further proof through randomized controlled trials is needed. This systematic review highlighted the importance of considering carotenoids as biological markers to monitor micronutrient status, providing evidence that strong recommendations to adopt increased fruit and vegetable intake may be part of the potential interventions to promote the prevention and better management of disability in the aging population.

## Figures and Tables

**Table 1:** Search strategy used in the US National Library of Medicine (PubMed) and Medical Literature Analysis and Retrieval System Online (MEDLINE) and adapted to the other sources, according to selected descriptors.

Strategy	Descriptors Used
# 1	(“frailty”[tiab]) OR (“frailty model”[tiab]) OR (“frailty phenotype”[tiab]) OR (“frailty syndrome”[tiab]) OR (fragility[tiab]) OR (physical performance[tiab]) OR (“grip”[tiab]) OR (“gait”[tiab])
# 2	(“caroten”[tiab]) OR (“micronutrient”[tiab]) OR (“α-carotene” [tiab]) OR (“β-carotene”[tiab]) OR (“lycopene”[tiab]) OR (“lutein”[tiab]) OR (“zeaxantin”[tiab]) OR (“β-cryptoxanthin”[tiab])
# 3	(Review) OR (Systematic review) OR (Narrative review) OR (Meta-analysis)
# 4	#1 AND #2 NOT #3

**Table 2:** Selected studies investigating carotenoids in relation to physical frailty (*n* = 11).

Author, Year (Ref.)	Country	Gender (%)	Carotenoids Exposure	Population	Design	*n*	Age (Years)	Outcome(s)	Findings
Semba RD et al., 2003 [33]	America (USA)	100%F	α-carotene β-carotene Total carotenoids Lutein/zeaxanthin	Women’s Health and Aging Studies (WHAS) I and II (Community-dwelling)	Cross-sectional	669	70–79	Grip strength Hip strength Knee strength	Higher plasma concentrations of α-carotene, β-carotene, β-cryptoxanthin, and lutein/zeaxanthin were associated with a reduced risk of low grip, hip, and knee strength.
Michelon E et al., 2006 [34]	America (USA)	100%F	α-carotene β-carotene Total carotenoids Lutein/zeaxanthin	Women’s Health and Aging Studies (WHAS) (Community-dwelling)	Cross-sectional	754	70–80	Physical Frailty	Low plasma concentrations of carotenoids (α-carotene, β-carotene, lutein/zeaxanthin, β-cryptoxanthin, and total carotenoids) were strongly associated with frailty.
Semba RD et al., 2006 [35]	America (USA)	100%F	Total carotenoids	Women’s Health and Aging Study I (WHAS I) (Community-dwelling)	Longitudinal, 3-year	766	65+	Physical Frailty	Women in the lowest quartile of total serum carotenoids had an increased risk of frailty over 3 years.
Smit E et al., 2013 [36]	America (USA)	NA	Total carotenoids	Third National Health and Nutrition Examination Survey (NHANES III) (non-institutionalized population)	Cross-sectional	4731	60+	Physical Frailty	Total serum carotenoids were significantly lower in the group with physical frailty compared with non-frail subjects.
Jayanama et al., 2018 [37]	America (USA)	52%F 48%M	α-carotene β-carotene Lycopene Lutein/zeaxanthin	Third National Health and Nutrition Examination Survey (NHANES) (non-institutionalized population)	Cross-sectional	9030	20+	Physical Frailty	High serum levels of α-carotene, β-carotene, β-cryptoxanthin, lutein/zeaxanthin, and lycopene were inversely associated with the Frailty Index (FI) score.
Rietman ML et al., 2018 [38]	Austria. Belgium Denmark. Finland France. Germany Greece. Italy the Netherlands Poland: Romania Spain. Switzerland United Kingdom	48.8%F 51.2%M	α-carotene β-carotene	The MARK-AGE Study (Community)	Cross-sectional	2128	35–74	Physical Frailty	Significantly lower levels of α-carotene, β-cryptoxanthin, and β-carotene were observed in physical frailty phenotypes compared with non-frail subjects.
Kochlik et al., 2019 [39]	Austria. France Germany. Italy Spain. Switzerland Sweden United Kingdom United States	55.9%F 44.1%M	α-carotene β-carotene Lycopene Lutein/zeaxanthin	FRAILOMIC (Community)	Cross-sectional	1450	65+	Physical Frailty	Frail participants were more likely to be classified in the lowest than in the highest tertile for α-carotene, βcarotene, lycopene, and β-cryptoxanthin than robust participants.
O’Halloran AM et al., 2019 [40]	Ireland (Europe)	51.3%F 48.7%M	Lutein Zeaxanthin	The Irish Longitudinal Study on Ageing (TILDA) (Community)	Cross-sectional	4068	50+	Physical Frailty	Mean concentrations of lutein and zeaxanthin were significantly, progressively, and consistently lower among the prefrail and frail groups across the CHS frailty phenotype, Frailty Index (FI), and FRAIL instruments.
Pilleron S et al., 2019 [41]	France Italy (Europe)	55.8%F 44.2%M	α-carotene β-carotene Lycopene Lutein/zeaxanthin	FRAILOMIC (Community)	Longitudinal, 2-year	221	65+	Physical Frailty	Total carotenoids, α-carotene, β-carotene, lycopene, cryptoxanthin, and lutein/zeaxanthin were significantly lower in the group with physical frailty in cross-sectional analyses. The 2-year prospective analysis confirmed significance only for total carotenoids and lutein/zeaxanthin.
Gomez-Cabrero D et al., 2021 [42]	Spain France Italy (Europe)	56%F 44%M	Serum Lutein/zeaxanthin	TSHA, InChianti, 3C-Bordeaux, AMI (Community)	Cross-sectional	1522	77–94	Physical Frailty	Lutein/zeaxanthin was found to be a protective marker against the frailty risk.
Sahni S et al., 2021 [43]	America (USA)	55%F 45%M	α-carotene β-carotene Total carotenoids Lutein/zeaxanthin	Framingham Heart Study Offspring cohort (FHS) (Community)	Longitudinal, 12-year	2452	33–88	Grip strength Gait speed	Daily intake of lycopene, lutein/zeaxanthin, and total carotenoids improves physical function in terms of either grip strength or walking speed. On improvement of walking speed, α-carotene and β-carotene were also found positive.

**Table 3:** Descriptive summary reporting differences in plasma carotenoid values by frailty status (presence/absence) across selected studies.

Author, Year (Ref.)	Carotenoids Exposure	Plasma Carotenoids Levels (mmol/L or mmol/L or mg/dL)		Outcome(s)
		Non-Frail	Frail	
Semba RD et al., 2003 [33]	α-carotene β-carotene Total carotenoids Lutein/zeaxanthin	NA	NA	Grip strength Hip strength Knee strength
Michelon E et al., 2006 [34]	α-carotene β-carotene Total carotenoids Lutein/zeaxanthin	0.097 (0.088–0.107) * 0.440 (0.401–0.485) * 1.842 (1.741–1.949) * 0.410 (0.388–0.434) * 0.136 (0.126–0.147) *	0.058 (0.048–0.070) * 0.296 (0.249–0.352) * 1.376 (1.249–1.515) * 0.323 (0.288–0.363) * 0.090 (0.077–0.106) *	Physical Frailty
Semba RD et al., 2006 [35]	Total carotenoids	1.48 (1.42–1.55) *	1.33 (1.25–1.42) *	Physical Frailty
Smit E et al., 2013 [36]	Total carotenoids	82.5 (1.5) †	75.1 (1.8) †	Physical Frailty
Jayanama et al., 2018 [37]	α-carotene β-carotene Lycopene Lutein/zeaxanthin	NA	NA	Physical Frailty
Rietman ML et al., 2018 [38]	α-carotene β-carotene	0.15 (0.09–0.25) § 0.58 (0.37–0.88) § 0.22 (0.12–0.38) §	0.10 (0.05–0.16) § 0.39 (0.26–0.55) § 0.15 (0.07–0.29) §	Physical Frailty
Kochlik et al., 2019 [39]	α-carotene β-carotene Lycopene Lutein/zeaxanthin	0.13 (0.12–0.14) * 0.44 (0.41–0.47) * 0.40 (0.37–0.42) * 0.36 (0.35–0.38) * 0.22 (0.21–0.24) *	0.11 (0.10–0.12) * 0.35 (0.32–0.39) * 0.29 (0.26–0.31) * 0.27 (0.25–0.28) * 0.16 (0.14–0.18) *	Physical Frailty
O’Halloran AM et al., 2019 [40]	Lutein Zeaxanthin	CHS frailty phenotype 0.198 (0.095) † 0.053 (0.032) †	CHS frailty phenotype 0.130 (0.067) † 0.032 (0.025) †	Physical Frailty
		Frailty Index (FI) 0.199 (0.093) 0.054 (0.033)	Frailty Index (FI) 0.199 (0.093) 0.054 (0.033)	
		FRAIL Scale 0.196 (0.094) 0.052 (0.032)	FRAIL Scale 0.196 (0.094) 0.052 (0.032)	
Pilleron S et al., 2019 [41]	α-carotene β-carotene Lycopene Lutein/zeaxanthin	132.5 (205.0) § 466.5 (535.0) § 389.0 (423.0) § 352.0 (228.0) § 219.0 (279.0) §	115.0 (187.0) § 387.5 (486.0) § 305.5 (380.0) § 267.5 (183.0) § 143.0 (234.0) §	Physical Frailty
Gomez-Cabrero D et al., 2021 [42]	Serum Lutein/zeaxanthin	NA	NA	Physical Frailty
Sahni S et al., 2021 [43]	α-carotene β-carotene Total carotenoids Lutein/zeaxanthin	NA	NA	Grip strength Gait speed

NA: not applicable, based on data provided by full-text article. * Data are expressed as means and 95% confidence intervals. † Data are expressed as means and standard deviation (SD). § Data are expressed as median (IQR).

**Table 4:** Summary of findings on the relationship between carotenoids and physical frailty.

Exposure	Evidence Base	Stenght of Association	Stenght of Evidence (GRADE)
Total carotenoids	Five studies	Logistic regression analysis between total plasma carotenoids by quartiles (lowest as a reference) and grip strength (OR: 0.37, 95% CI 0.21–0.65 [33] Logistic regression analysis between total plasma carotenoids by quartiles (lowest as a reference) and hip strength (OR 0.31, 95% CI 0.17–0.54) [33] Logistic regression analysis between total plasma carotenoids by quartiles (lowest as a reference) and knee strength (OR 0.44, 95% CI 0.26–0.75) [33] Logistic regression analysis between plasma total carotenoids (μmol/L) by quartiles (highest quartile as reference) and frailty risk (OR 2.50; 95% CI 1.51–4.14) [34] Compared with subjects in the upper three quartiles, women in the lowest quartile of plasma carotenoids (hazard ratio [HR] 1.39; 95% CI 1.01–1.92) had an increased risk of becoming frail over 3 years [35] Plasma levels of carotenoids were significantly lower in people who were frail compared with non-frail (*p* = 0.01) [36] Regression analysis between total carotenoids intake (for 10 mg higher intake/d) and annualized change in grip strength (kg/y): positive regression coefficient of 0.0316, *p* = 0.03 [43] Regression analysis between total carotenoids intake (for 10 mg higher intake/d) and annualized change in gait speed (m/s per year) (kg/y): positive regression coefficient of 0.0021, *p* < 0.01 [43]	⊕ ⊕ ⊕ Moderate
α-carotene	Seven studies	Logistic regression analysis between plasma α-carotene by quartiles (lowest as a reference) and grip strength (OR 0.30, 95% CI 0.17–0.52) [33] Logistic regression analysis between plasma α-carotene by quartiles (lowest as a reference) and hip strength (OR 0.28, 95% CI 0.16–0.48) [33] Logistic regression analysis between plasma α-carotene by quartiles (lowest as a reference) and knee strength (OR 0.38, 95% CI 0.22–0.65) [33] Logistic regression analysis between plasma α-carotene (μmol/L) by quartiles (highest quartile as reference) and frailty risk (OR 2.24; 95% CI 1.34–3.74) [34] Regression analysis between low plasma α-carotene (<1.3 μmol/L) and frailty: positive regression coefficient of 0.023, 95% CI 0.018–0.028, *p* < 0.001 [37] Analysis on physical frailty and plasma α-carotene (μmol/L) using Rank-ANOVA showed significantly low levels in the frail, *p* = 0.0078 [38] Logistic regression analysis between plasma α-carotene (μmol/L) by tertiles (highest tertile as reference) and frailty risk (OR 1.69; 95% CI 1.00–2.88) [39] Plasma α-carotene was significantly lower in the group with physical frailty, *p* < 0.001 [41] Regression analysis between α-carotene intake (for 10 mg higher intake/d) and annualized change in gait speed (m/s per year) (kg/y): positive regression coefficient of 0.0187, *p* = 0.02 [43]	⊕ ⊕ ⊕⊕ High
β-carotene	Seven studies	Logistic regression analysis between plasma β-carotene by quartiles (lowest as a reference) and grip strength (OR 0.34, 95% CI 0.20–0.60) [33] Logistic regression analysis between plasma β-carotene by quartiles (lowest as a reference) and hip strength (OR 0.36, 95% CI 0.21–0.62) [33] Logistic regression analysis between plasma β-carotene by quartiles (lowest as a reference) and knee strength (OR 0.47, 95% CI 0.28–0.79) [33] Logistic regression analysis between plasma β-carotene (μmol/L) by quartiles (highest quartile as reference) and frailty risk (OR 1.84; 95% CI 1.13–2.99) [34] Regression analysis between low plasma β-carotene (<6.4 μmol/L) and frailty: positive regression coefficient of 0.025, 95% CI 0.020–0.030), *p* < 0.001 [37] Analysis on physical frailty and plasma β-Carotene (μmol/L) using Rank-ANOVA showed significantly low levels in the frail, *p* = 0.0242 [38] Logistic regression analysis between plasma β-carotene (μmol/L) by tertiles (highest tertile as reference) and frailty risk (OR 1.84; 95% CI 1.13–2.99) [39] Plasma β-carotene was significantly lower in the group with physical frailty, *p* < 0.001 [41] Regression analysis between β-carotene intake (for 10 mg higher intake/d) and annualized change in gait speed (m/s per year) (kg/y): positive regression coefficient of 0.0080, *p* < 0.01 [43]	⊕ ⊕ ⊕⊕ High
β-cryptoxanthin	Five studies	Logistic regression analysis between plasma β-cryptoxanthin (μmol/L) by quartiles (highest quartile as reference) and frailty risk (OR 2.34; 95% CI 1.38–3.99) [33] Logistic regression analysis between plasma β-cryptoxanthin by quartiles (lowest as a reference) and grip strength (OR 0.52, 95% CI 0.30–0.90) [33] Logistic regression analysis between plasma β-cryptoxanthin by quartiles (lowest as a reference) and hip strength (OR 0.41, 95% CI 0.24–0.70) [33] Logistic regression analysis between plasma β-cryptoxanthin by quartiles (lowest as a reference) and knee strength (OR 0.54, 95% CI 0.32–0.91) [33] Regression analysis between low plasma β-cryptoxanthin (<4.0 μmol/L) and frailty: positive regression coefficient of 0.031, 95% CI 0.026–0.036, *p* < 0.001 [37] Analysis on physical frailty and plasma β-cryptoxanthin (μmol/L) using Rank-ANOVA showed significantly low levels in the frail, *p* = 0.0130 [38] Logistic regression analysis between plasma β-cryptoxanthin (μmol/L) by tertiles (highest tertile as reference) and frailty risk (OR 3.02; 95% CI 1.95–4.69) [39] Plasma β-cryptoxanthin was significantly lower in the group with physical frailty, *p* < 0.001 [41]	⊕ ⊕ ⊕ Moderate
Lycopene	Four studies	Regression analysis between low plasma lycopene (<11.9 μmol/L) and frailty: positive regression coefficient of 0.022, 95% CI 0.01–0.027, *p* < 0.001 [37] Logistic regression analysis between plasma lycopene (μmol/L) by tertiles (highest tertile as reference) and frailty risk (OR 1.94; 95% CI 1.24–3.05) [39] Plasma lycopene was significantly lower in the group with physical frailty, *p* < 0.001 [41] Regression analysis between lycopene intake (for 10 mg higher intake/d) and annualized change in grip strength (kg/y): positive regression coefficient of 0.0873, *p* < 0.01 [43] Regression analysis between lycopene intake (for 10 mg higher intake/d) and annualized change in gait speed (m/s per year) (kg/y): positive regression coefficient of 0.0043, *p* < 0.01 [43]	⊕ ⊕ ⊕ Moderate
Lutein/zeaxanthin	Seven studies	Logistic regression analysis between plasma lutein/zeaxanthin (μmol/L) by quartiles (highest quartile as reference) and frailty risk (OR 2.92; 95% CI 1.75–4.88) [34] Regression analysis between low plasma lutein/zeaxanthin (<11.1 μmol/L) and frailty: positive regression coefficient of 0.032, 95% CI 0.028–0.036, *p* < 0.001 [37] Logistic regression analysis between plasma lutein/zeaxanthin (μmol/L) by tertiles (highest tertile as reference) and frailty risk (OR 3.60; 95% CI 2.34–5.53) [39] Logistic regression analysis between plasma lutein (μmol/L) and the risk of pre-frailty (relative risk ratios (RRRs): 0.78–0.86) and frailty (RRRs: 0.43–0.63) [40] Logistic regression analysis between plasma zeaxanthin (μmol/L) and the risk of pre-frailty (RRRs: 0.79–0.87) and frailty (RRRs: 0.49–0.63) [40] Prospective 2-year analysis maintained significance only for plasma lutein/zeaxanthin (*p* < 0.02) [41] Logistic regression analysis between plasma lutein/zeaxanthin (μmol/L) and frailty risk (OR: 0.82, 95% CI: 0.70–0.97) [42] Regression analysis between lutein/zeaxanthin intake (for 10 mg higher intake/d) and annualized change in grip strength (kg/y): positive regression coefficient of 0.1223, *p* = 0.04 [43] Regression analysis between lutein/zeaxanthin intake (for 10 mg higher intake/d) and annualized change in gait speed (m/s per year) (kg/y): positive regression coefficient of 0.0084, *p* < 0.01 [43] Logistic regression analysis between plasma lutein/zeaxanthin by quartiles (lowest as a reference) and grip strength (OR 0.29, 95% CI 0.17–0.50) [43] Logistic regression analysis between plasma lutein/zeaxanthin by quartiles (lowest as a reference) and hip strength (OR 0.26, 95% CI 0.15–0.46) [43] Logistic regression analysis between plasma lutein/zeaxanthin by quartiles (lowest as a reference) and knee strength (OR 0.39, 95% CI 0.22–0.68) [43]	⊕ ⊕ ⊕⊕ High

## Data Availability

The original contributions presented in the study are included in the article, further inquiries can be directed to the corresponding authors.
